# Effect of predatory bacteria on the gut bacterial microbiota in rats

**DOI:** 10.1038/srep43483

**Published:** 2017-03-06

**Authors:** Kenneth Shatzkes, Chi Tang, Eric Singleton, Sean Shukla, Michael Zuena, Shilpi Gupta, Sonal Dharani, Joseph Rinaggio, Nancy D. Connell, Daniel E. Kadouri

**Affiliations:** 1 Division of Infectious Disease, Department of Medicine, Rutgers New Jersey Medical School, Newark, NJ 07103, USA; 2 Department of Oral Biology, Rutgers School of Dental Medicine, Newark, NJ 07103, USA; 3 Department of Diagnostic Sciences, Rutgers School of Dental Medicine, Newark, NJ 07103, USA

## Abstract

*Bdellovibrio bacteriovorus* and *Micavibrio aeruginosavorus* are Gram-negative proteobacteria that are obligate predators of other Gram-negative bacteria and are considered potential alternatives to antibiotics. Most studies focusing on predatory bacteria have been performed *in vitro*, thus the effect of predatory bacteria on a live host, including the impact on the ecology of the native microbiota, has yet to be fully examined. In this study, intrarectal inoculations of Sprague-Dawley rats with predatory bacteria were performed. Additionally, feces were collected for seven days post-inoculation to determine the effect on gut bacterial diversity. Rat colonic tissue exhibited no abnormal histopathological effects due to predatory bacteria. A modest increase in pro-inflammatory cytokines was measured in the colons of rats inoculated with predatory bacteria by 24 and 48 hours, with all but IL-13 returning to baseline by seven days. V4 16S rRNA gene sequencing of fecal DNA demonstrated minimal shifts in taxonomic representation over the week due to predatory bacteria. Changes in bacterial populations due to exposure to *B. bacteriovorus* are predicted to contribute to health, however, an overgrowth of *Prevotella* was observed due to exposure to *M. aeruginosavorus*. This study further addresses safety concerns associated with the potential use of predatory bacteria to treat infections.

With the rise of multi-drug resistant (MDR) bacterial infections over the last decade^1^, researchers have begun to develop new ways to treat infections for the future post-antibiotic era^2^. One alternative therapy currently being explored is the use of predatory bacteria^3^. *Bdellovibrio bacteriovorus* and *Micavibrio aeruginosavorus* are Gram-negative Delta- and Alphaproteobacteria, respectively, and are obligate predators of other Gram-negative bacteria^4^,^5^. *B. bacteriovorus* use a type IV pilus to attach to a prey cell, then invades across the cell membrane to establish itself in the periplasm of the prey forming a bdelloplast, before digesting the prey cell from within^6^,^7^,^8^. When the nutrients are exhausted, the growing *B. bacteriovorus* will divide, lyse the bdelloplast, and then continue to seek new prey. In contrast, *M. aeruginosavorus* are ‘vampire-like’ bacteria, as they attach to the prey cell outer membrane to leach nutrients from the outside^5^,^9^,^10^.

Predatory bacteria present several advantages that promote their potential use to combat bacterial infection. The effectiveness of both *B. bacteriovorus* and *M. aeruginosavorus* to limit the growth of key human pathogens, including those causing MDR infections^11^, *in vitro* is well documented^3^,^12^,^13^,^14^. Unlike with the use of traditional antibiotics, development of genetically-stable resistance to predation has yet to be confirmed^15^. Predatory bacteria are also obligate bacterial predators, shown not to harm many mammalian cell lines^16^,^17^,^18^. However, most studies focusing on predatory bacteria have been performed using *in vitro* methodologies, and thus the full effect of introducing predatory bacteria into a live host has yet to be determined.

The safety of administering predatory bacteria *in vivo* has already been examined in multiple animal models, such as mice, rats, rabbits, guinea pigs, and chicks^3^,^19^,^20^,^21^,^22^,^23^. It was recently demonstrated that intranasal or intravenous inoculation of high doses of either *B. bacteriovorus* or *M. aeruginosavorus* in mice resulted in no apparent pathological effects and induced a modest immune response that returned to physiological levels by 24 hours post-inoculation^20^. Additionally, both predators were not detected in animal tissues by 48 hours post-inoculation, potentially removed by innate immune mechanisms^20^. While the safety of introducing predatory bacteria into an animal host through respiratory and intravenous routes has already been assessed, we are interested in the effect predatory bacteria would have on the immune system within the gastrointestinal tract and on its residing commensal bacterial microbiota. This inquiry is of interest, not only because predatory bacteria have the capability to control bacterial populations, but also because evidence has suggested that *B. bacteriovorus* may actually contribute to health by being a member of the healthy human gut microbiota^24^.

As *B. bacteriovorus* might not survive within the acidic environment of the stomach^19^, administering predatory bacteria through oral gavage would not allow us to assess the effect they would have within the colon. To this end, intrarectal inoculations of rats with predatory bacteria were performed in this study to determine the effect on the gut immune response and the commensal bacterial microbiota populations. The work presented here further addresses some of the safety concerns associated with the use of predatory bacteria as a novel anti-microbial therapeutic.

## Results

### Host Morbidity

**Figure 1:**
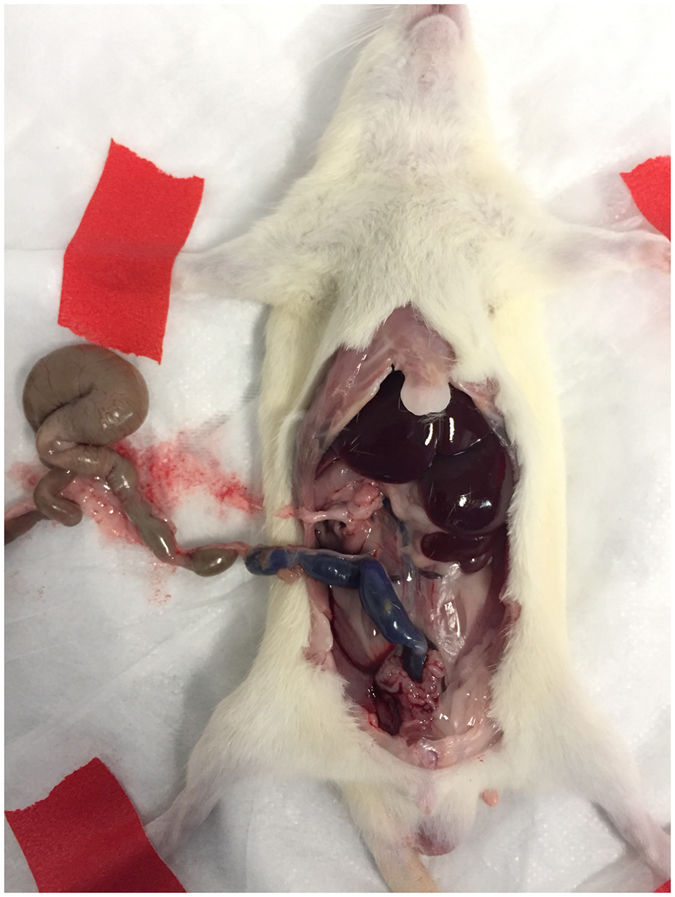
Intrarectal inoculation of predatory bacteria into rats. Predatory bacteria were introduced by intracolonic instillation of Sprague Dawley rats. Animals were anaesthetized with 4% isoflurane in oxygen for five minutes. A 3Fr polyurethane catheter with a 1 mL syringe attached via a Luer Lock was inserted intrarectally to approximately 8 cm proximal to the anus, at which 250 μl of bacterial suspension (diluted in PBS) was slowly injected into the colon. In this image, Crystal Violet was inoculated to visualize where the inoculums would travel within the colon.

To examine the effect of introducing predatory bacteria into the gut on host morbidity, we inoculated 8.2 × 10^8^ plaque forming units (PFU)/rat of *B. bacteriovorus* 109J or 4.6 × 10^7^ PFU/rat of *M. aeruginosavorus* ARL-13 into the colons of two groups of Sprague Dawley (SD) rats (64 rats each) via a rectal catheter (Fig. 1). Two more groups of rats were inoculated with the vehicle, phosphate buffered saline (PBS; 64 rats), or sub-lethal concentrations of 9.4 × 10^7^ CFU/rat of *Klebsiella pneumoniae* ATCC 43826 (16 rats), a known pathogen. The aforementioned animals and treatment groups were used for all subsequent experiments described in this study. Rats were visually monitored for up to seven days for any signs of infection, illness or discomfort. At seven days post-inoculation, all 192 rats that were inoculated with PBS or with predatory bacteria over the course of the experiment were healthy and showed no adverse effects. Overall, our data indicate that introducing predatory bacteria into the gut have no effect on SD rat host morbidity.

### Histological Examination

**Figure 2:**
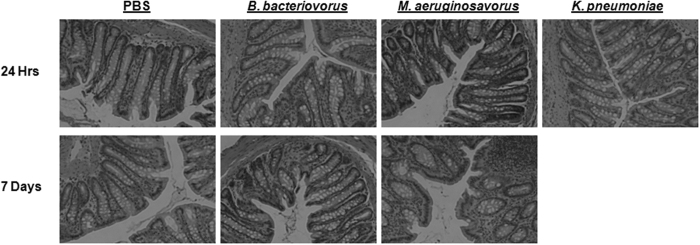
Histological examination of distal colons from rats after intrarectal inoculation of predatory bacteria. PBS, *B. bacteriovorus, M. aeruginosavorus*, or *K. pneumoniae* were introduced into the colons of SD rats via intrarectal inoculation. Histological examination of rat colons revealed no pathological abnormalities compared to rats inoculated with the control, PBS. All images are representative micrographs taken at 24 hours and 7 days post-inoculation and at 40X total magnification.

To determine if predatory bacteria can cause detrimental histopathological effects within the colon, histological examination of colon tissue was performed on the previously described SD rats inoculated with PBS, *B. bacteriovorus, M. aeruginosavorus*, or *K. pneumoniae*, sacrificed at three, 24, 48 hours or seven days post-inoculation. Examination was performed by a pathologist blinded to each specimen’s treatment group, and findings were similar across all groups. Cross-sections of harvested colons were lined by a simple columnar epithelium and contained numerous evenly spaced glands (Fig. 2). Occasional mitotic figures were seen and the lamina propria and submucosa contained scattered lymphocytes and eosinophils (Fig. 2). Deposits of mucosa-associated lymphoid tissue were seen in some specimens (Fig. 2). Some specimens exhibited focal mucosal erosion, however, this was not accompanied by an observed inflammatory reaction and was most likely due to post-mortem mechanical abrasion. No other abnormalities were noted, indicating that introducing predatory bacteria into the rat gut results in no visually abnormal pathological effect.

### Host Immune Response

**Figure 3:**
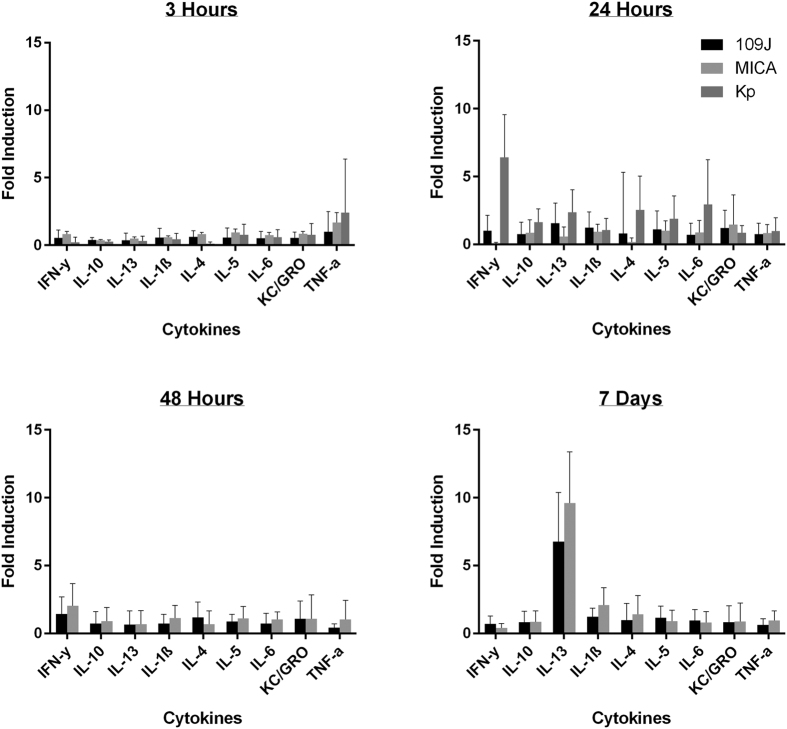
Inflammatory protein profile within rat colons in response to intrarectal inoculation of predatory bacteria. ELISA analysis of IL-1β, IL-4, IL-5, IL-6, IL-10, IL-13, CXCL-1/KC, IFNγ, and TNF in response to intrarectal inoculation of predatory bacteria relative to PBS control. *B. bacteriovorus* (109J), *M. aeruginosavorus* ARL-13 (MICA) or *K. pneumoniae* (Kp) as a control were introduced into the colons of SD rats via intrarectal inoculation. Inflammatory proteins were assessed within the colon at 1, 24, 48 hours and 7 days post-inoculation. Sixteen rats per treatment group (eight for *K. pneumoniae*) were used at each time point. Data is combined from two independent experiments. Data represent mean ± standard error of the mean.

To examine the effects of predatory bacteria on the rat gut immune response, the previously described rat groups were sacrificed at three, 24, 48 hours and seven days post-inoculation (16 rats/time point for the PBS and predatory bacteria groups; eight rats/time point for *K. pneumoniae*) when distal colon samples were harvested for ELISA. At three, 24 and 48 hours, none of the rats exposed to any of the predatory bacteria strains exhibited significant increases in any inflammatory protein assayed (Fig. 3). A 6.8- and 9.6-fold increase in IL-13 in rats exposed to *B. bacteriovorus* and *M. aeruginosavorus*, respectively, was measured at seven days post-inoculation, however, none of the other inflammatory cytokines demonstrated substantial change compared to control at that time point (Fig. 3). Rats inoculated with *K. pneumoniae* demonstrated higher inductions of most inflammatory cytokines tested (particularly a 6.4-fold increase in IFNγ) by 24 hours post-inoculation (Fig. 3). In comparison, rats inoculated with *B. bacteriovorus* and *M. aeruginosavorus* demonstrated no change and a 10-fold decrease in IFNγ, respectively (Fig. 3). Collectively, our data indicate that *B. bacteriovorus* and *M. aeruginosavorus* do not provoke a robust inflammatory response within the gastrointestinal tract.

### Whole Bacterial Microbiota Overview

To examine the effect of predatory bacteria on the gut commensal bacterial microbiota, feces were collected daily for seven days from five rats from each treatment group. Sequencing of the V4 region of the 16S rRNA gene was performed on DNA extracted from feces in order to determine if there were significant changes in the bacterial communities in the gut. 16S rRNA gene sequencing produced 8,110,049 quality-filtered reads, giving a mean sample depth of 81,100 reads. Sequence reads per sample ranged from 23,302 to a maximum of 263,152 filtered reads.

**Figure 4:**
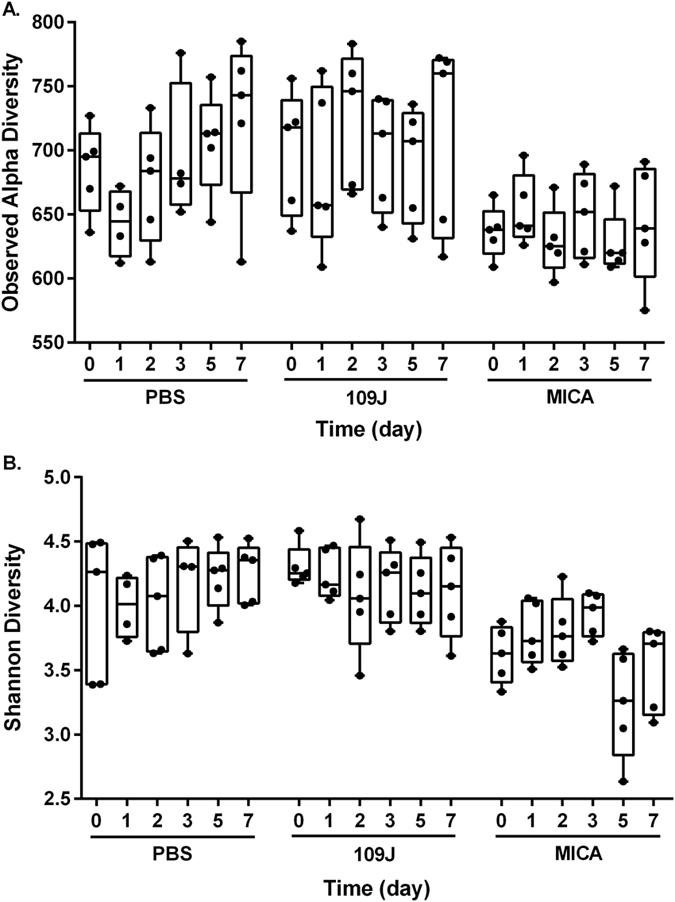
Alpha-diversity estimates. Observed alpha and Shannon diversity of microbiota samples tested from feces collected from rats intrarectally inoculated with PBS, *B. bacteriovorus* (109J) or *M. aeruginosavorus* (MICA) over seven days. Points represent diversity measures of each individual rat. Horizontal lines represent means.

**Figure 5:**
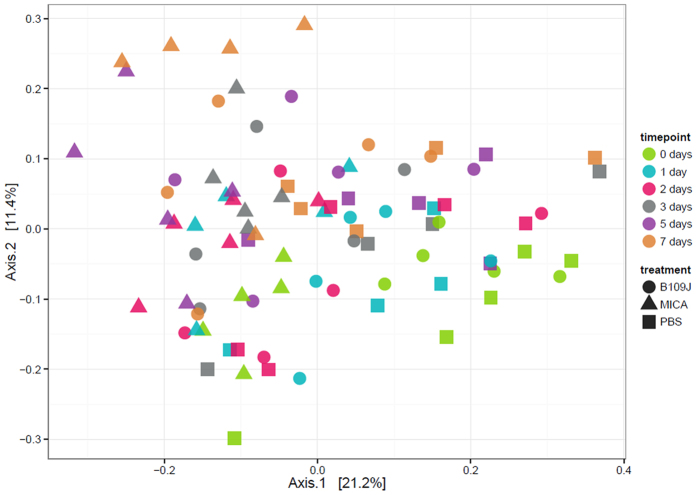
Weighted ordination (beta diversity). Dimensional reduction of the Bray-Curtis distance between all microbiota samples tested from feces collected from rats intrarectally inoculated with PBS, *B. bacteriovorus* (B109J) or *M. aeruginosavorus* (MICA) over seven days, using the principal coordinate analysis (PCoA) ordination method.

At week’s end, linear regression analysis determined there was no significant difference in the observed or Shannon diversity (alpha diversity) for either *B. bacteriovorus*-treated or *M. aeruginosavorus*-treated samples compared to pre-treatment (Fig. 4). *M. aeruginosavorus*-treated samples had significantly less alpha diversity than both PBS and *B. bacteriovorus*-treated samples before treatment at day 0 (Fig. 4). Additionally, samples appeared to separate slightly according to day feces were collected in weighted ordination analysis, which compares community composition, of all samples sequenced (Fig. 5). PERMANOVA found time point (*p* < 0.002) and cage number which the animal was housed (*p* < 0.001) significantly contributed to bacterial microbiota beta diversity differences. Samples also clustered by treatment and sub-clustered according to cage number in hierarchical clustering analysis (Supp. Fig. S1).

### Bacterial Microbiota Changes Over Time

**Figure 6:**
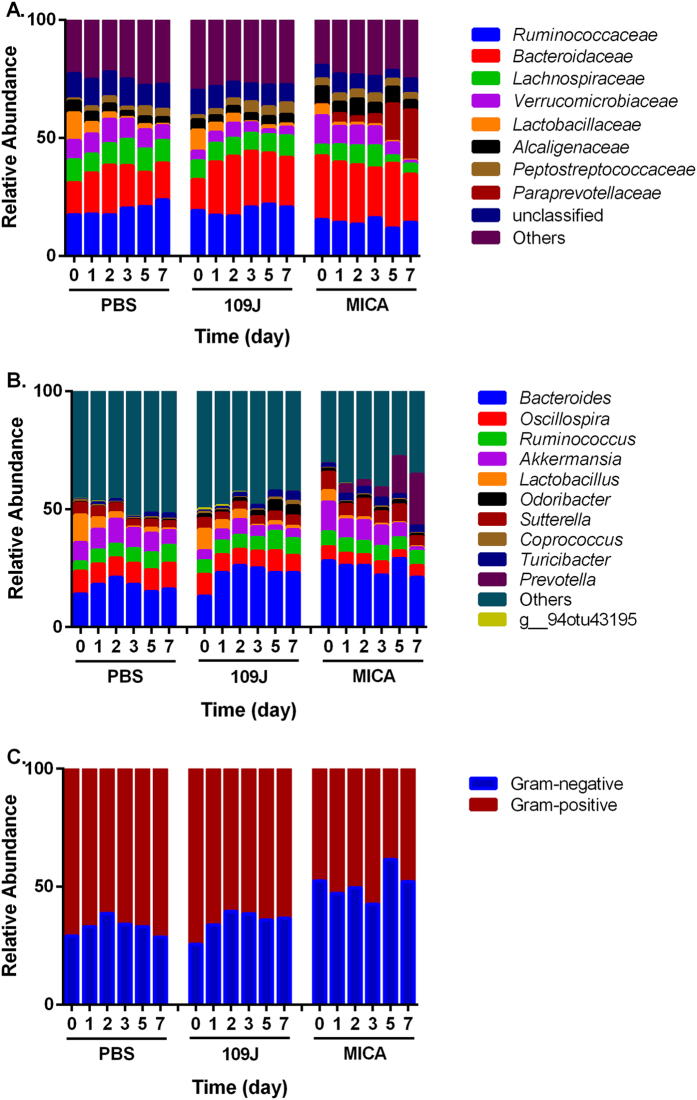
Proportional taxa abundance over time. Mean relative abundances of most abundant microbial populations by (**A**) Family, (**B**) Genus, and (**C**) Gram-status in feces collected from rats intrarectally inoculated with PBS, *B. bacteriovorus* (109J) or *M. aeruginosavorus* (MICA) over seven days. Gram status was generally assigned based on phylum. Abundances by individual rat are available in the Supplemental information.

The changes in gut bacterial populations due to each individual treatment over time were analyzed. In samples inoculated with the vehicle, PBS, genus *Lactobacillus* (and family *Lactobacillaceae*) relative abundance significantly decreased nearly 17-fold (*p* = 0.007), while families *Ruminococcaceae* and *Peptostreptococcaceae* relative abundance increased 1.4- (*p* = 0.011) and 3.7-fold (*p* = 0.002), respectively over time (Fig. 6A,B, Supp. Figs S2A and S3A). No significant shifts or concordant patterns were observed in the Gram-negative to Gram-positive bacterial ratios present in the microbiota (Fig. 6C, Supp. Fig. S4A). Bacterial populations slightly shifted according to the day feces were collected in weighted ordination analysis (*p* = 0.001; Supp. Fig. S5A) and samples clustered according to cage number in hierarchical clustering analysis (Supp. Fig. S6). There were no significantly differential operational taxonomic units (OTUs) detected out of 904 features tested. OTUs did not have a significantly large enough shift in relative abundance (absolute value of log_2_ fold change greater than one) over time.

In animals inoculated with *B. bacteriovorus*, genus *Coprococcus* significantly increased 2.2-fold (*p* = 0.01) in relative abundance (family *Peptostreptococcaceae* increased 2.7-fold, *p* = 0.01) and *Lactobacillus* significantly decreased 6.4-fold (*p* = 0.001) over the seven days (*Lactobacillaceae* increased 6.7-fold, *p* = 0.01; Fig. 6A,B, Supp. Figs S2B and S3B). While a mean 11% shift in the abundance from Gram-positive to Gram-negative bacteria was detected at day 7, there was high variation between individual rats (Fig. 6C, Supp. Fig. S4B). In an attempt to detect *B. bacteriovorus* in our sequencing reads, we detected four OTUs belonging to the Deltaproteobacteria class (*Desulfovibrio, Bilophila*, and two unclassified members of the *Desulfovibrionaceae* family and Myxococcales order). Additionally, the relative abundance of Deltaproteobacteria in the microbiota decreased on average nearly 4.0-fold at two days post-inoculation before increasing over the rest of the week. Principal coordinate analysis (PCoA) plots showed that bacterial populations appeared to shift according to the time feces were collected and formed two main clusters correlating with time point: one cluster was associated with the earlier time points of 0–2 days, and a second cluster with 3–7 days (*p* = 0.001; Supp. Fig. S5B). Samples further sub-clustered according to cage number according to hierarchical clustering analysis (Supp. Fig. S7). The bacterial communities in the rat gut were relatively stable after inoculation with predatory bacteria over time. Only two OTUs (out of 911 features tested), both belonging to Firmicutes, were significantly differentially abundant. *Coprococcus eutactus* had a 1.13 log_2_ fold increase per day (*p* < 0.05) and an unidentified member of Clostridiales had a 1.03 log_2_ fold decrease per day (*p* < 0.05).

In rats inoculated with *M. aeruginosavorus*, genus *Akkermansia* (family *Verrucomicrobiaceae*) relative abundance significantly decreased over 9.2-fold (*p* = 0.0002) and genus *Prevotella* (family *Paraprevotellaceae*) significantly increased 219-fold (*p* = 0.0004) over time (Fig. 6A,B, Supp. Figs S2C and S3C). As with the other treatments, *Lactobacillus* relative abundance also decreased 8.5-fold compared to pre-treatment (Fig. 6B). No significant shifts were detected in Gram-negative to Gram-positive bacterial ratios present in the microbiota (Fig. 6C, Supp. Fig. S4C). When attempting to detect *M. aeruginosavorus* in our sequencing reads, there did not appear to be a concordant trend in Alphaproteobacteria over time, however, 12 OTUs were identified as Alphaproteobacteria, including two which had 89% and 84% matches to *M. aeruginosavorus* ARL-13. Populations appeared to shift along the coordinate axes in weighted ordination analysis (*p* = 0.001; Supp. Fig. S5C and S8). Again, bacterial communities were relatively stable, as only one OTU (out of 910 features tested), from *Prevotella*, had a significant 1.09 log_2_ fold increase per day (*p* < 0.05).

### Bacterial Microbiota Changes Due to Predatory Bacteria

**Figure 7:**
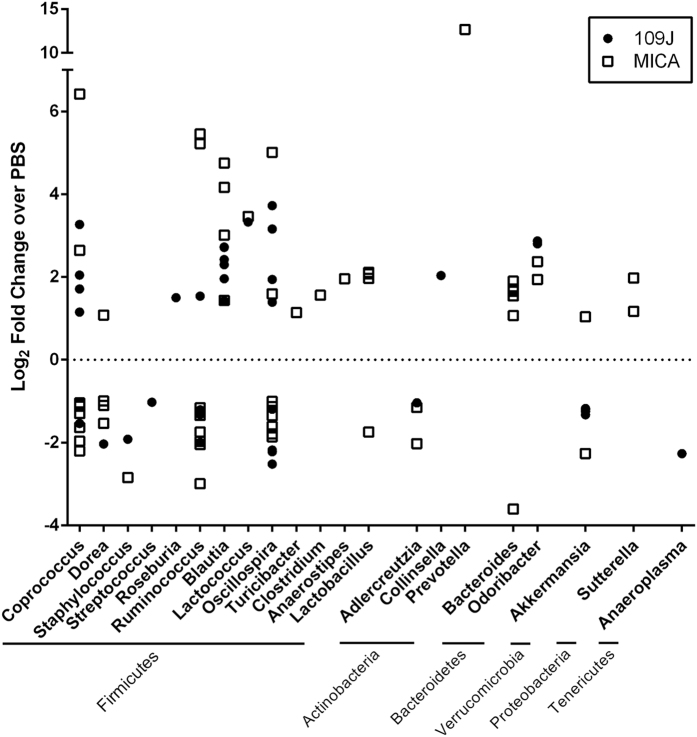
Differentially abundant features. Differentially abundant features of *B. bacteriovorus* (109J) and *M. aeruginosavorus*-treated (MICA) samples compared to PBS. Each point represents an OTU belonging to the respective genus. Corresponding phyla are listed below genera. Features were considered significant if their FDR-corrected *p*-value was less than or equal to 0.05, and the absolute value of the log_2_ fold change was greater or equal to 1. Note that unclassified/unnamed OTUs at the genus level were omitted.

The microbiota population changes due to inoculation with predatory bacteria compared to PBS control was determined next. Significantly, *B. bacteriovorus*-treated samples had 1.9-fold less *Verrucomicrobiaceae* than PBS samples (*p* < 0.001; Supp. Fig. S9A). In PCoA plots, *B. bacteriovorus*-treated samples clustered by time point and along both coordinate axes according to the day feces were collected (*p* = 0.001; Supp. Fig. S10A). Hierarchical clustering analysis showed samples also clustered according to cage number and treatment group (Supp. Fig. S11). There were a total of 166 differentially abundant OTUs (out of 911 features tested). Eighty-six OTUs, 77 of which were Firmicutes, were significantly overabundant in samples treated with *B. bacteriovorus* compared to PBS (*p* < 0.05; Fig. 7). Classified taxa at the genus level that were significantly overabundant in *B. bacteriovorus*-treated samples (n = OTU count) include *Blautia* (n = 6), *Collinsella* (n = 1), *Coprococcus* (n = 4), *Lactococcus* (n = 1), *Odoribacter* (n = 2), *Oscollospira* (n = 4), *Roseburia* (n = 1), and *Ruminococcus* (n = 1). An additional 69 OTUs identified as Firmicutes were also significantly reduced in *B. bacteriovorus*-treated samples (*p* < 0.05; Fig. 7). Genera that were found to be significantly reduced in *B. bacteriovorus*-treated samples compared to PBS include *Adlercreutzia* (n = 1), *Akkermansia* (n = 4), *Anaeroplasma* (n = 1), *Coprococcus* (n = 1), *Dorea* (n = 1), *Oscillospira* (n = 4), *Ruminococcus* (n = 3), *Staphylococcus* (n = 1), and *Streptococcus* (n = 1). Interestingly, OTUs with some of the highest log_2_ fold reduction were Gram-positive genera.

*M. aeruginosavorus*-treated samples had significantly higher relative abundances of *Bacteroidaceae, Alcaligenaceae*, and *Paraprevotellacaeae* than PBS-treated samples (*p* < 0.001; Supp. Fig. S9B). Significantly lower relative abundances of *Ruminococcaceae, Lachnospiraceae* and an unclassified family from Clostridiales were also observed in *M. aeruginosavorus*-treated samples compared to PBS (*p* < 0.001; Fig. 6B). *M. aeruginosavorus*-treated bacterial microbiotas clustered and shifted along the coordinate axes according to treatment in weighted ordination analysis (*p* = 0.001; Supp. Fig. S10B). Samples also clustered primarily by treatment, then by cage number, in hierarchical clustering analysis (Supp. Fig. S12). There were a total of 280 differentially abundant OTUs (out of 912 features tested), 122 of which were overabundant in *M. aeruginosavorus*-treated samples compared to PBS (*p* < 0.05; Fig. 7). Taxa at the genus level that were significantly overabundant in *M. aeruginosavorus*-treated samples include *Akkermansia* (n = 1), *Anaerostipes* (n = 1), *Bacteroides* (n = 7), *Blautia* (n = 4), *Clostridium* (n = 1), *Coprococcus* (n = 2), *Dorea* (n = 1), *Lactobacillus* (n = 3), *Lactococcus* (n = 1), *Odoribacter* (n = 2), *Oscillospira* (n = 2), *Prevotella* (n = 1), *Ruminococcus* (n = 2), *Sutterella* (n = 2), and *Turicibacter* (n = 1). 153 OTUs from Firmicutes were also found to be significantly reduced in *M. aeruginosavorus*-treated samples (*p* < 0.05; Fig. 7). Genera that were found to be significantly reduced in samples treated with *M. aeruginosavorus* compared to PBS include *Adlercreutzia* (n = 2), *Akkermansia* (n = 1), *Bacteroides* (n = 1), *Coprococcus* (n = 7), *Dorea* (n = 3), *Lactobacillus* (n = 1), *Oscillospira* (n = 6), *Ruminococcus* (n = 2), and *Staphylococcus* (n = 1). Once again, some of the OTUs that had the highest log_2_ fold reduction were from Gram-positive genera. Overall, the data show that there were minimal shifts in the bacterial communities over the course of the experiment, indicating that introducing predatory bacteria into the gastrointestinal tract of rats does not have a significant impact on the gut bacterial populations based on 16 S rRNA gene sequence analysis.

## Discussion

With the limited amount of new antibiotics being developed, scientists are looking for innovative ways to combat bacterial infection^2^, one of which is predatory bacteria. While the ability of predatory bacteria to control human pathogens *in vitro* is well documented, questions remain regarding the safety of introducing bacterial predators into a mammal. Here, our study focuses on the effect of administering predatory bacteria into the gastrointestinal tract of mammals.

We began by investigating the effect of introducing predatory bacteria into the gastrointestinal tract on rat morbidity, histopathology of the gut and its immune response. We intrarectally inoculated rats with PBS, predatory bacteria or *K. pneumoniae* and sacrificed animals at three, 24, 48 hours and seven days to harvest organs for analysis. The dose concentration of predatory bacteria administered was similar to our previous study examining the safety and efficacy of predatory bacteria within rat lungs^23^, however, the volume at which the dose was administered was increased from 50 μl to 250 μl in order to cover the larger surface area of the colon. *K. pneumoniae* is a Gram-negative opportunistic pathogen responsible for many hospital-acquired infections that can readily colonize the human gastrointestinal tract^25^, and while any Gram-negative bacterium could have been used as a comparison in this study, we sought to use a clinically relevant strain known to cause disease within the gut. A sub-lethal dose of *K. pneumoniae* was used in order to ensure rats survived the length of the experiment. All 128 rats inoculated with predatory bacteria into their gastrointestinal tracts were healthy with no visual adverse effects. Histology of the gut revealed no apparent inflammation or pathological effects due to predatory bacteria. Furthermore, none of the rats exposed to any of the predatory bacteria strains exhibited significant increases in any inflammatory protein assayed by 48 hours post-inoculation.

These results are consistent with previous studies that have demonstrated that predatory bacteria are non-toxic in different animal models and induce only a modest inflammatory response^19^,^20^,^21^,^22^. Interestingly, IL-13 levels remained elevated at day seven in rats inoculated with *B. bacteriovorus* or *M. aeruginosavorus*. Levels of IL-13 were slightly higher in rats inoculated with *M. aeruginosavorus* than with *B. bacteriovorus* at day seven, possibly explained by the fact *M. aeruginosavorus* are epibiotic predators and are potentially more easily accessible by the immune response elements. IL-13 has anti-inflammatory properties and is associated primarily with the induction of disease in the airways^26^,^27^,^28^, however, studies have shown IL-13 secreted by Th2 cells induce changes that are required to remove intracellular parasites from the mouse gut^29^,^30^,^31^. IL-13 can also regulate matrix metalloproteinases to protect against excessive inflammation in many tissues^32^, and it is possible that physiological changes in the gut due to predatory bacteria are being corrected through similar mechanisms. Future studies will focus on further understanding how the immune response specifically reacts to remove predatory bacteria from the gut environment.

We next looked to determine the effect of introducing predatory bacteria into the gut on the commensal microbiota over time. Samples inoculated with PBS showed slight shifts as time progressed. Specifically, a significant decrease in *Lactobacillus*, a Gram-positive genus, was seen across all treatment groups, including PBS, over time, signaling administration of PBS itself may have induced some minor microbiota changes. However, in samples inoculated with *M. aeruginosavorus*, this decrease in *Lactobacillus* was accompanied with a significant increase of *Prevotella*, which is a Gram-negative genus. An overgrowth of *Prevotella* and a reduction of *Lactobacillus* have been previously correlated with the onset of osteomyelitis in mice^33^. Interestingly, a previous study looking at the effect of *B. bacteriovorus* HD100 on multispecies oral communities found the periplasmic predator able to prey on *Prevotella intermedia* in co-cultures, but not in multispecies models^34^. In addition, a decrease in *Akkermansia*, a Gram-negative genus, was observed. Studies have shown *Akkermansia* to be present in higher abundances in healthy subjects compared to those with inflammatory bowel syndrome^35^, obesity and type 2 diabetes^36^. Although the measured changes in the bacterial microbiota might signal a potential negative outcome due to introducing *M. aeruginosavorus* into the gut, no changes in animal well-being or behavior was observed.

In samples inoculated with *B. bacteriovorus*, there was only one significant differential OTU which was an increase in *Coprococcus eutactus*, a Gram-positive genus. A previous study found decreased abundances of *Coprococcus eutactus* in inflammatory bowel syndrome (IBS) patients suffering with severe IBS-related symptoms^37^. *B. bacteriovorus* treatment may be able to increase *Coprococcus eutactus* abundances in these patients, potentially reversing microbiota dysbiosis. Overall, there were minimal shifts in other bacterial communities within each treatment over the seven days due to inoculation with predatory bacteria.

While we were unable conclusively to detect *B. bacteriovorus* in our fecal samples, we did detect four OTUs matching Deltaproteobacteria. In addition, a decrease in Deltaproteobacteria was seen at two days post-inoculation, however, populations significantly increased from that point forward for the rest of the week. One might suggest that the changes in Deltaproteobacteria relative abundance over the week could be the result of *B. bacteriovorus* predation early in the week before bacterial communities began to stabilize. A previous study was also not able to detect *B. bacteriovorus* in the fecal and cecal contents of young chicks after oral administration of predatory bacteria (and an antacid), signaling that exogenous *B. bacteriovorus* introduced into the gut environment may be short-lived^19^. However, it is important to note the relative abundance of Deltaproteobacteria is very low in the context of the whole bacterial microbiota community (assuming the rat gut contains ~10^13^ bacterial cells, the final ratio of predatory bacteria to commensal bacteria was approximately one in 100,000), and sequencing at a deeper depth will be required to fully understand these changes at a finer taxonomic level. Similar to our own results, the same chick study also observed an average ~10-fold decrease in *Lactobacillus* abundances in chick cecal contents after oral administration of *B. bacteriovorus* HD100^19^. We were able more conclusively to detect *M. aeruginosavorus* in our samples, as we found 12 OTUs matching to Alphaproteobacteria, two of which had high homology to *M. aeruginosavorus*.

Interestingly, there is evidence that predatory bacteria may already be present in healthy human flora. Multiple studies have detected *B. bacteriovorus* within the gut^22^,^24^,^38^,^39^ and oral^40^ microbiomes of humans and other animals. A recent study argued that further introducing bacterial predators back into the gut microbiota may alleviate some of the diseases associated with dysbiosis and loss of microbial diversity in the western world^41^. *B. bacteriovorus* populations are governed by the Lotka-Volterra prey-predator oscillation^42^, where increases in *B. bacteriovorus* abundances are balanced by phenotypic resistance to predation by the prey^15^. Therefore, it could be predicted that *B. bacteriovorus* present in the gastrointestinal tract may act as an ecological balancer of gut microbial populations contributing to health. In fact, a study found *B. bacteriovorus* are present in higher abundance within the gastrointestinal tract of healthy individuals compared to patients with Inflammatory Bowel Diseases, Celiac disease and Cystic fibrosis^24^.

It was hypothesized that since *B. bacteriovorus* and *M. aeruginosavorus* are obligate Gram-negative predators, they would shift the abundance between Gram-positive and Gram-negative bacteria over time. We did not observe any significant shift or concordant pattern in the Gram-negative to Gram-positive bacterial ratios present in the microbiota due to either *B. bacteriovorus* or *M. aeruginosavorus* treatment. We determined 166 and 280 differentially abundant OTUs between *B. bacteriovorus* or *M. aeruginosavorus*-treated samples, respectively, and PBS control. Interestingly, the majority of classified OTUs either significantly reduced or increased by inoculation with predatory bacteria belong to Firmicutes, a largely Gram-positive phylum. A possible explanation for this result is that predatory bacteria are not directly reducing Gram-positive bacteria, but rather microbiota changes in the gastrointestinal tract brought about due to the presence of predatory bacteria could allow certain bacterial populations to displace other populations, regardless of whether they are Gram-negative or Gram-positive genera. The previous study that orally administered predatory bacteria to young chicks found that while *Lactobacillus* populations in the gastrointestinal tract significantly decreased in abundance, *Streptococcus* populations increased in abundance^19^, signaling that changes in the gut microbiota cannot simply be predicted based on whether the bacterial prey of interest is Gram-negative or Gram-positive. Interestingly, in our own study, the only significantly differential OTU representing the *Streptococcus* genus was found to be reduced in samples treated with *B. bacteriovorus*. It is also possible that since Firmicutes are the dominant phyla in the gut bacterial microbiota, it is easier to detect changes in these populations due to sheer relative abundance compared to other phyla. Population changes in other phyla occupying the gastrointestinal tract may be detected if sequencing depth is increased in future studies.

Some of the other OTUs with the highest log_2_ fold increases due to either *B. bacteriovorus* or *M. aeruginosavorus* compared to PBS belonged to the genus *Blautia. Blautia* is a Gram-positive genus associated with normal health flora and has been shown to be one of the members of the gut microbiota to play an important role in recovery from *Vibrio cholerae* infection in mice^43^. Thus, it is possible that the overabundance in *Blautia* is in direct response to the introduction of *Bdellovibrio* and *Micavibrio* into the gut environment. Among genera that contained OTUs that were both increased and decreased due to predatory bacteria exposure were *Coprococcus* and *Oscillospira. Oscillospira* is a Gram-positive genus that has never been cultured in the laboratory, but is associated with leanness and health^44^. While it is unlikely that these two Gram-positive genera are being preyed upon, differential rates of predation by *B. bacteriovorus* and *M. aeruginosavorus* on species within the same genus have been observed before^11^,^12^. Thus, it is not surprising to see many genera belonging to the same taxonomic family being affected differently due to predatory bacteria inoculation.

It is important to view the changes in the bacterial microbiota due to inoculation of predatory bacteria in the context of population shifts brought about by antibiotics. Antibiotics, while an efficient treatment for infection, have off-target effects that can cause dysbiosis within the gut microbiome, often leading to significant decreases in bacterial diversity^45^,^46^,^47^,^48^,^49^. In fact, a study demonstrated that changes in the gut microbiota due to antibiotic use may last for up to a year before returning to previous abundances^50^. Furthermore, the same study demonstrated that exposure to different antibiotics enriched genes associated with antibiotic resistance^50^. In contrast, *B. bacteriovorus* minimally shifted the bacterial microbiota as compared to antibiotics, and with research beginning to demonstrate a link between health and normal gastrointestinal flora, this represents a major advantage if predatory bacteria are to be developed into a new treatment for infection.

In conclusion, our results suggest that introducing predatory bacteria into the rat gut does not result in any measurable adverse pathological effects and does not cause a substantial immune response within the gut. Furthermore, the limited changes in gut bacterial populations due to intrarectal inoculation of *B. bacteriovorus* directly into the gastrointestinal tract were mostly associated with healthy benefits, while potential adverse negative effects were seen in microbiota changes due to *M. aeruginosavorus*. One could suggest that increasing the number of doses and the concentration of predatory bacteria may result in more substantial changes in the bacterial microbiota, and future studies will address this. The limited population shifts in the microbiota due to predatory bacteria compares favorably against the off-target effects of antibiotics. With the increased prevalence of antibiotic resistant infections, this study provides further support for developing predatory bacteria into a novel antimicrobial treatment. Future studies will focus on further understanding how predatory bacteria are removed from the gut by the immune response, as well as increasing sequencing depth to further decipher changes in the microbiota at the species level due to administration of predatory bacteria.

## Methods

### Bacterial strains and growth conditions

Bacterial strains used in this study were *Bdellovibrio bacteriovorus* 109 J (ATCC 43826), *Micavibrio aeruginosavorus* strain ARL-13^10^, and *Klebsiella pneumoniae* ATCC 43816. *K. pneumoniae* was cultured in LB media. Predatory bacteria were cultured and processed as previously described^17^. In brief, predator stock lysates were made by co-culturing the predators with *Escherichia coli* WM3064 used as prey, and incubating at 30 °C on a rotary shaker for 24 (*B. bacteriovorus*) and 72 hours (*M. aeruginosavorus*) until the cultures cleared. Fresh predator cultures were prepared from the stock lysates to obtain higher concentration of predators for the experiments, as described previously^17^. The fresh co-cultures were filtered twice through a 0.45-μm Millex pore-size filter (Millipore) and further purified and concentrated by three sequential centrifugations at 29,000 g for 45 minutes using a Sorvall LYNX 4000 centrifuge (Thermo Fisher Scientific Inc). The pellet was washed and re-suspended in 50 mL of PBS after each cycle. A plaque-forming unit (PFU) value between ~5 × 10^9^ to 5 × 10^10^ PFU/mL for *B. bacteriovorus* and ~5 × 10^8^ to 5 × 10^9^ PFU/mL for *M. aeruginosavorus*, was obtained when the final predator pellet was re-suspended in 1–2 mL of PBS solution to reach a final optical density (OD_600_) of 0.2 ± 0.02 for *B. bacteriovorus* and 0.1 ± 0.02 for *M. aeruginosavorus.* Fifty μl aliquots of predator samples were plated on DAP-supplemented LB agar, nutrient agar and TSB-blood plates to ensure that the samples were free from contamination and prey cells.

### Rats

Wild-type 4–6 week old male Sprague Dawley (SD) rats were purchased from Charles River Laboratories (Wilmington, MA, USA). A maximum of four rats were housed per cage. All rats were housed under pathogen-free conditions at the Rutgers New Jersey Medical School animal facility. All experiments were performed in accordance with the protocols approved by the Institutional Animal Care and Use Committee of Rutgers New Jersey Medical School (protocol #15012) and the Animal Care and Use Review Office of the U.S. Army Medical Research and Material Command were followed in handling the animals.

### Intrarectal Inoculation

Predatory bacteria were introduced by intracolonic instillation^51^ of Sprague Dawley (SD) rats. Animals were anaesthetized with 4% isoflurane in oxygen for five minutes. A 3Fr polyurethane catheter (Access Technologies, Skokie, Illinois, USA) with a 1 mL syringe attached via a Luer Lock was inserted intrarectally to approximately 8 cm proximal to the anus, at which 250 μl of bacterial suspension (diluted in PBS) was slowly injected into the colon. Rats were introduced with either PBS (vehicle), *B. bacteriovorus* 109J, *M. aeruginosavorus* ARL-13, or *K. pneumoniae* ATCC 43826. To prevent leakage of the bacterial suspension, animals were held at an angle with anus pinched closed for 30 seconds after removing the catheter from the colon. A smaller number of rats were used in the *K. pneumoniae* group in order to limit the number of animals being sacrificed. Being mindful of animal wellbeing, we also did not keep any rats inoculated with *K. pneumoniae* past 24 hours post-inoculation. After initial inoculation, animals were observed and visually assessed for signs of infection, illness and discomfort. To assess the immune response, rats were sacrificed at three, 24, 48 hrs and 7 days post-exposure, when colon samples were collected. Samples were used for histological examination and host immune response profiling (ELISA).

### Histological Examination

Distal colon samples were stored in formalin at 4 °C before examination. All histopathological examination was performed by a pathologist blinded to each specimen’s treatment group. Formalin-fixed organ segments from infected rats were paraffin-embedded and stained with hematoxylin and eosin (H&E) for cellular composition as previously described. Stained sections were analyzed and photographed using an EVOS FL Cell Imaging System (Life Technologies, Carlsbad, CA).

### Host Immune Response Profiling (ELISA)

Samples were prepared as previously described^20^. Cytokines were measured using a V-Plex Proinflammatory Panel2 (rat) Kit (K15059D-1); Meso Scale Discovery) according to manufacturer’s instructions, and read on a SECTOR Imager 2400 (Meso Scale Discovery).

### Fecal Collection and DNA Extraction

To determine the effect of predatory bacteria on the commensal gut bacterial microbiota, feces were collected from five rats from each treatment group for each day for up to seven days post-instillation and frozen immediately at −80 °C. DNA from feces was extracted using the PowerFecal DNA Isolation Kit (Mo Bio Laboratories). DNA quantity and quality was assessed using an Agilent 2100 Bioanalyzer (Santa Clara, CA, USA).

### Library Preparation

Purified DNA was sent to Second Genome Solutions (South San Francisco, CA, USA) for library preparation and sequencing. On arrival, all samples were quantified via the Qubit^®^ Quant-iT dsDNA High Sensitivity Kit (Invitrogen, Life Technologies, Grand Island, NY) to ensure that they met minimum concentration and mass of DNA. To enrich the sample for bacterial 16S V4 rRNA genes, DNA was amplified utilizing fusion primers designed against the surrounding conserved regions which are tailed with sequences to incorporate Illumina (San Diego, CA, USA) adapters and indexing barcodes. Each sample was PCR amplified with two differently bar-coded V4 fusion primers. Samples that met post-PCR quantification minimums were advanced for pooling and sequencing. For each sample, amplified products were concentrated using a solid-phase reversible immobilization method for the purification of PCR products and quantified by qPCR.

### Commensal Microbiota Profiling

A pool containing 16S V4 enriched, bar-coded samples were loaded into a MiSeq^®^ reagent cartridge, and then onto the instrument along with the flow cell. After cluster formation on the MiSeq instrument, the amplicons were sequenced for 250 cycles with custom primers designed for paired-end sequencing. Sequenced paired-end reads were merged, quality filtered, and de-replicated with USEARCH^52^. Resulting unique sequences were then clustered at 97% similarity by UPARSE and a representative consensus sequence per *de novo* OTU was determined. Sequences that passed quality filtering were then mapped to a set of representative consensus sequences to generate an OTU abundance table. Representative OTU sequences were assigned taxonomic classification via Mothur’s bayesian classifier at 80% confidence; the classifier was trained against the Greengenes^53^ reference database of 16S rRNA gene sequences clustered at 99%.

### Statistical Analysis

For ELISA, significant differences between treatment groups and respective control were analyzed using ANOVA. Alpha-diversity differences between beginning and end of treatment were compared using linear regression, controlling for animal ID. PERMANOVA^54^ (paired by animal ID) using distance matrices and was performed for each variable of interest to determine if they significantly contributed to the beta-diversity of the samples. Abundance-weighted sample pair-wise differences were calculated using Bray-Curtis^55^ dissimilarity and plotted using the Principal Coordinate Analysis (PCoA). Hierarchical clustering was done using the Ward 2 method. Differences in mean population abundances over time in the microbiota were compared using linear regression, controlling for animal ID. Univariate differential abundance of OTUs was tested using the DESeq2 package^56^. DESeq2 (paired by animal ID) was run under default settings and *q*-values were calculated with the Benjamin-Hochberg^57^ procedure to correct *p*-values, controlling for false discovery rates (FDR). OTUs were considered significant if their FDR-corrected *p*-value was less than or equal to 0.05, and the absolute value of the log_2_ fold change was greater than or equal to 1.

## Additional Information

**How to cite this article**: Shatzkes, K. *et al*. Effect of predatory bacteria on the gut bacterial microbiota in rats. *Sci. Rep.* **7**, 43483; doi: 10.1038/srep43483 (2017).

**Publisher's note:** Springer Nature remains neutral with regard to jurisdictional claims in published maps and institutional affiliations.

## Supplementary Material

Supplementary Information
