# Supplementary material for: Effect of predatory bacteria on the gut bacterial microbiota in rats
Source: Sci Rep. 2017 Mar 6;7:43483. doi: 10.1038/srep43483 (PMC5337950; doi:10.1038/srep43483)
Supplement: Supplementary Information [file srep43483-s1.pdf]

Supplemental Information for

Effect of predatory bacteria on the gut bacterial microbiota in rats

Kenneth Shatzkes[1], Chi Tang[1], Eric Singleton[1], Sean Shukla[1], Michael Zuena[1], Shilpi Gupta[2], Sonal Dharani[2], Joseph Rinaggio[3], Nancy D. Connell[1], and Daniel E. Kadouri[2*]

[1] Division of Infectious Disease, Department of Medicine, Rutgers New Jersey Medical School, Newark, NJ 07103, USA

[2] Department of Oral Biology, Rutgers School of Dental Medicine, Newark, NJ 07103, USA

[3] Department of Diagnostic Sciences, Rutgers School of Dental Medicine, Newark, NJ 07103, USA

* Address correspondence to Daniel E. Kadouri, kadourde@sdm.rutgers.edu

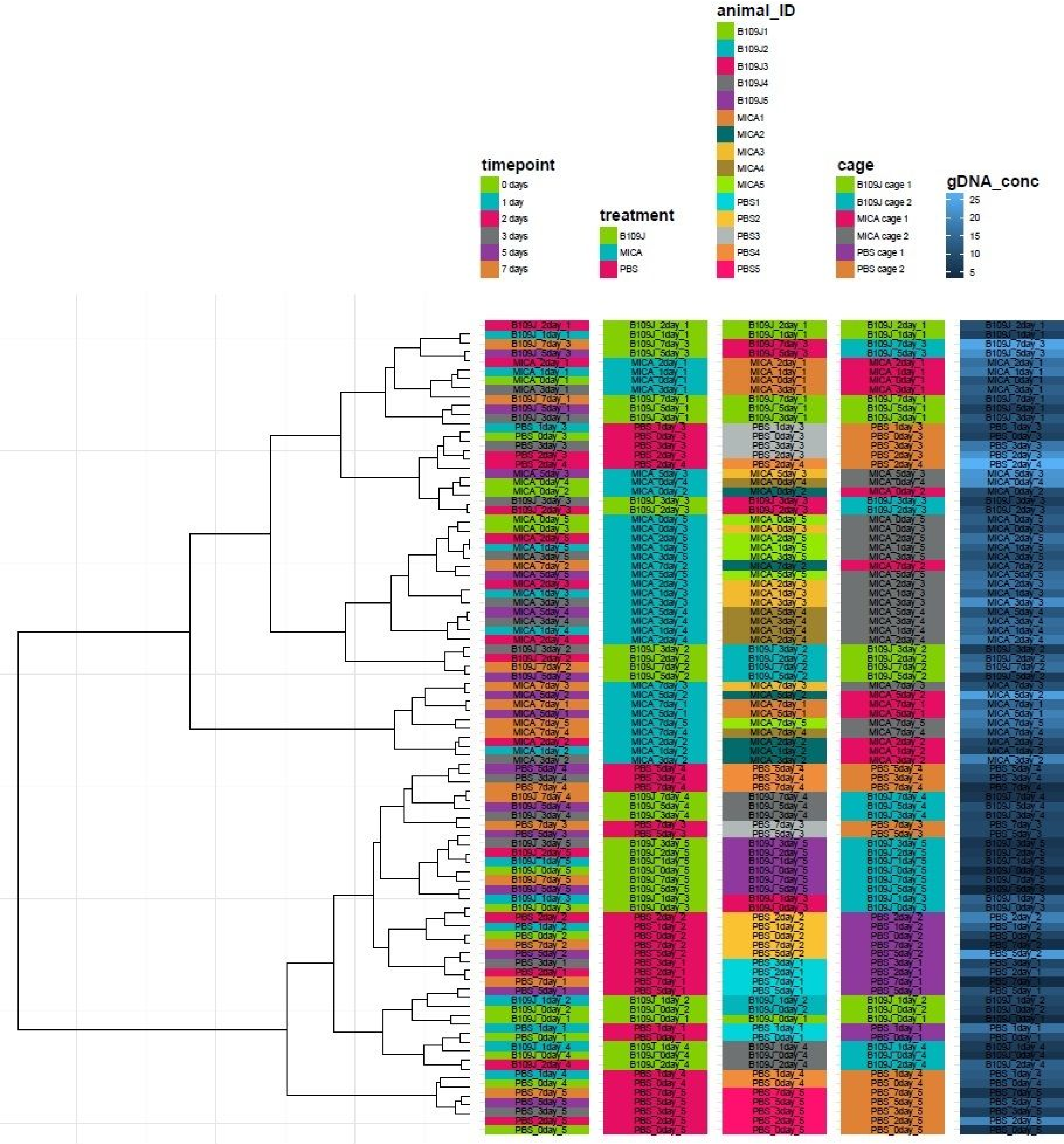

Supplementary Figure S1

Hierarchical clustering of all microbiota samples. Dendogram of all microbiota samples extracted from feces of rats intrarectally inoculated with PBS, *B. bacteriovorus* (109J), or *M. aeruginosavorus* (MICA). Dendograms were created using the Ward 2 method. Samples clustered by treatment, cage number, and animal ID. Sample name format is: Treatment_DayFecesCollected_AnimalID.

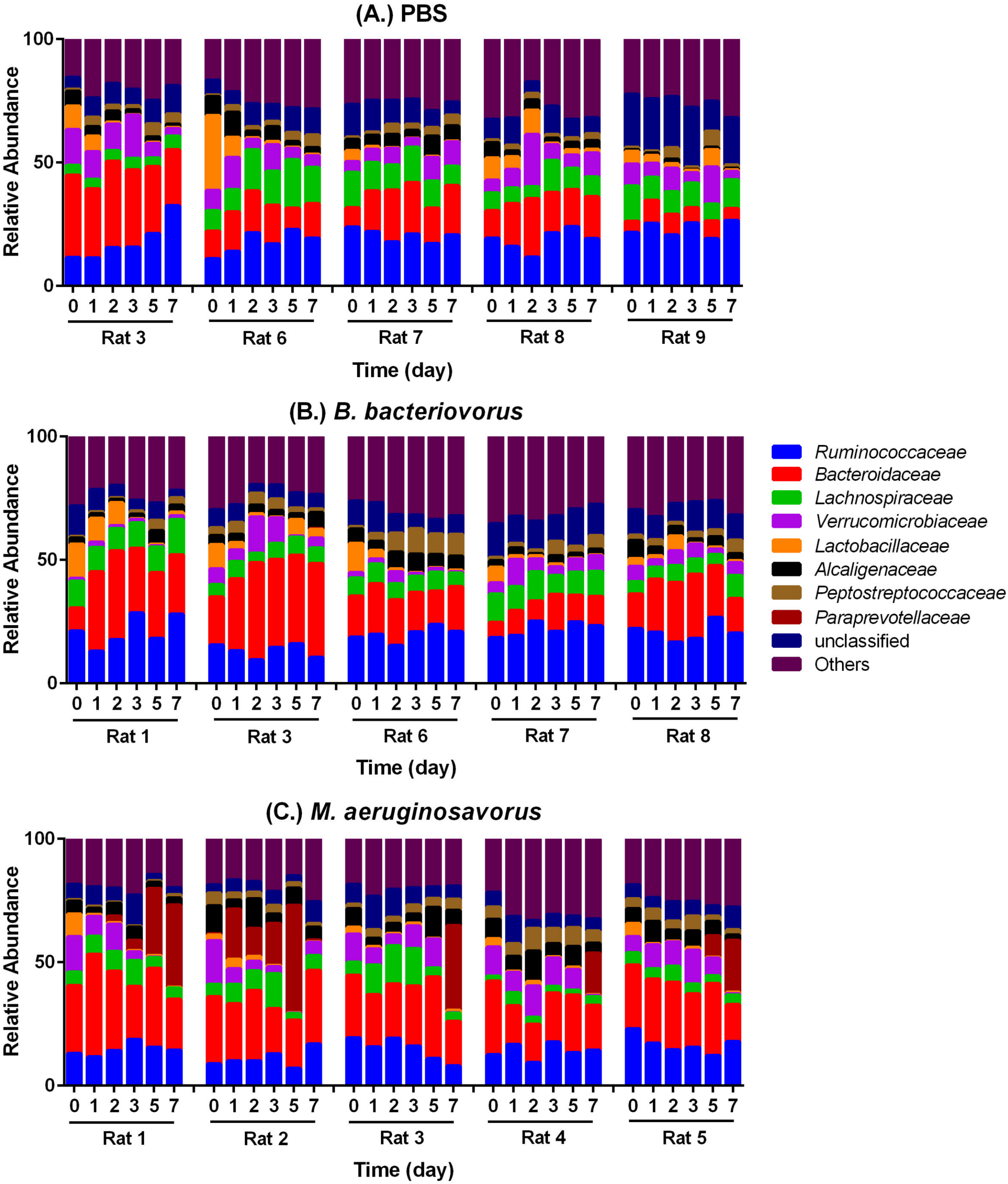

<u>Supplementary Figure S2</u>

Proportional family taxa abundance over time. Relative abundances of most abundant microbial populations at family level in feces collected from all rats intrarectally inoculated with (A) PBS, (B) *B. bacteriovorus* or (C) *M. aeruginosavorus* over seven days.

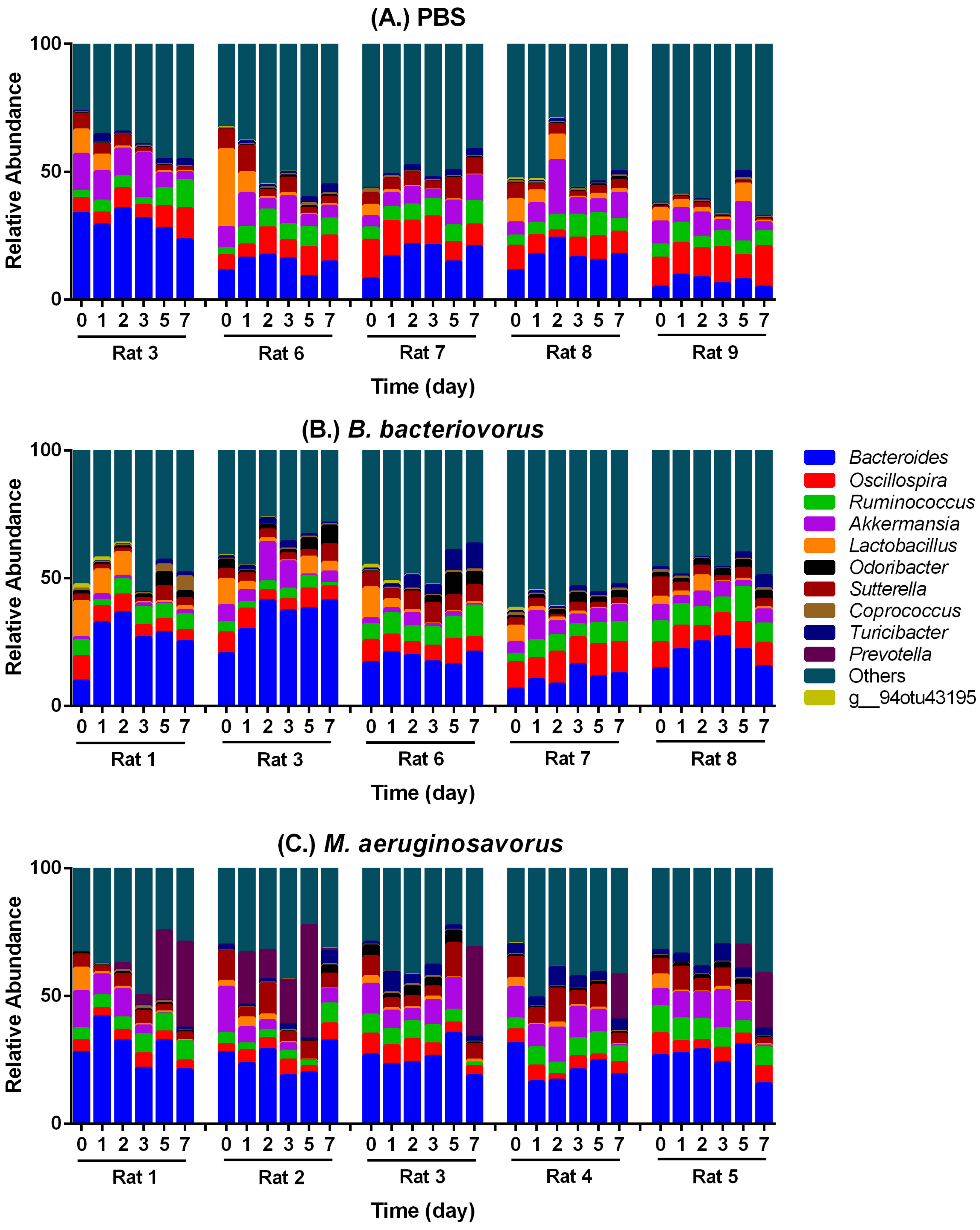

Supplementary Figure S3

Proportional genus taxa abundance over time. Relative abundances of most abundant microbial populations at genus level in feces collected from all rats intrarectally inoculated with (A) PBS, (B) *B. bacteriovorus* or (C) *M. aeruginosavorus* over seven days.

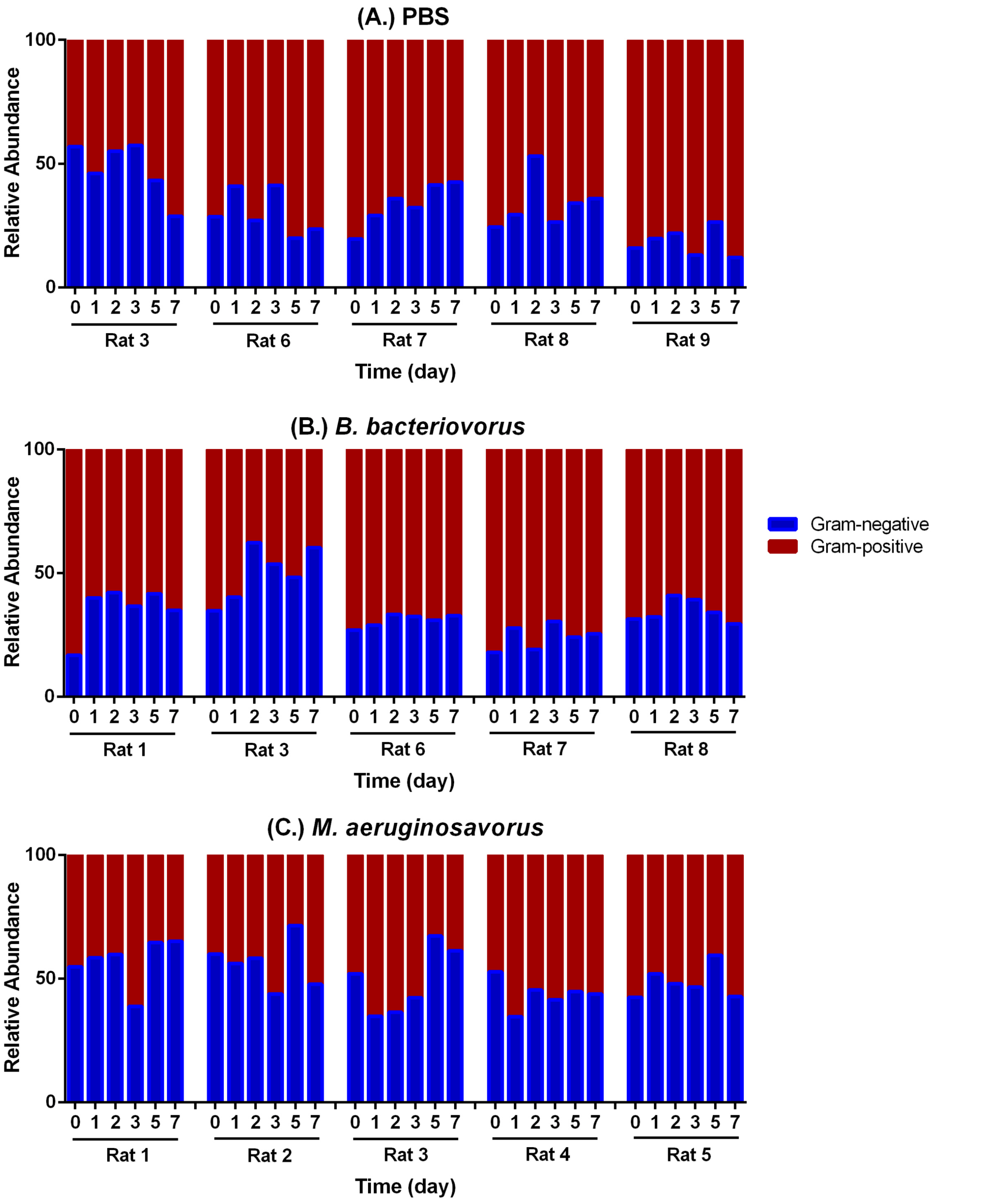

Supplementary Figure S4

Proportional abundance by Gram-status over time. Relative abundances by Gram-status in feces collected from all rats intrarectally inoculated with (A) PBS, (B) *B. bacteriovorus* or (C) *M. aeruginosavorus* over seven days.

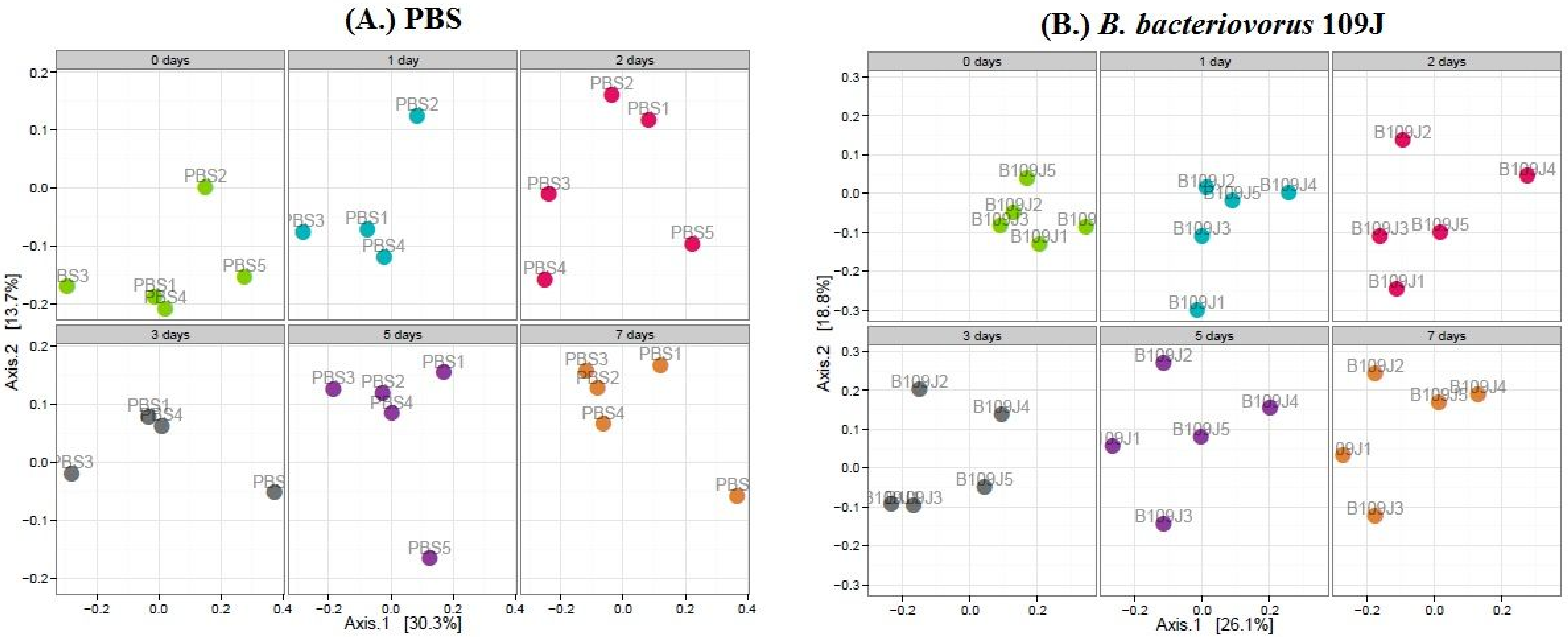

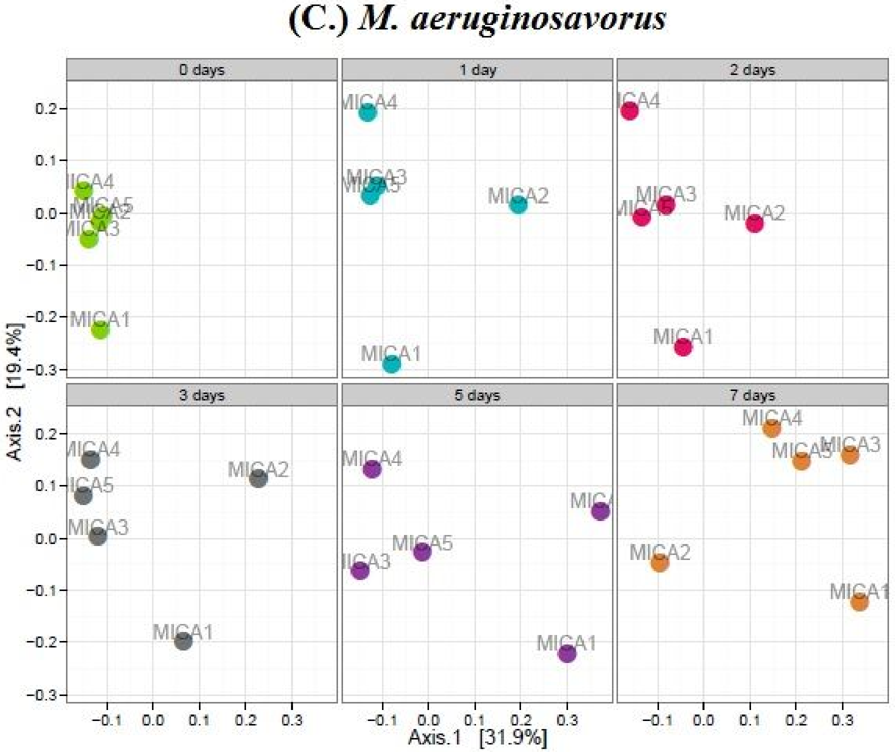

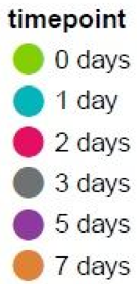

<u>**Supplementary Figure S5**</u>

Weighted ordination analysis of microbiota samples by treatment over time. Dimensional reduction of the Bray-Curtis distance between samples tested from feces collected from rats intrarectally inoculated with (A) PBS, (B) *B. bacteriovorus* (B109J) or (C) *M. aeruginosavorus* (MICA) over seven days, using the principal coordinate analysis (PCoA) ordination method.

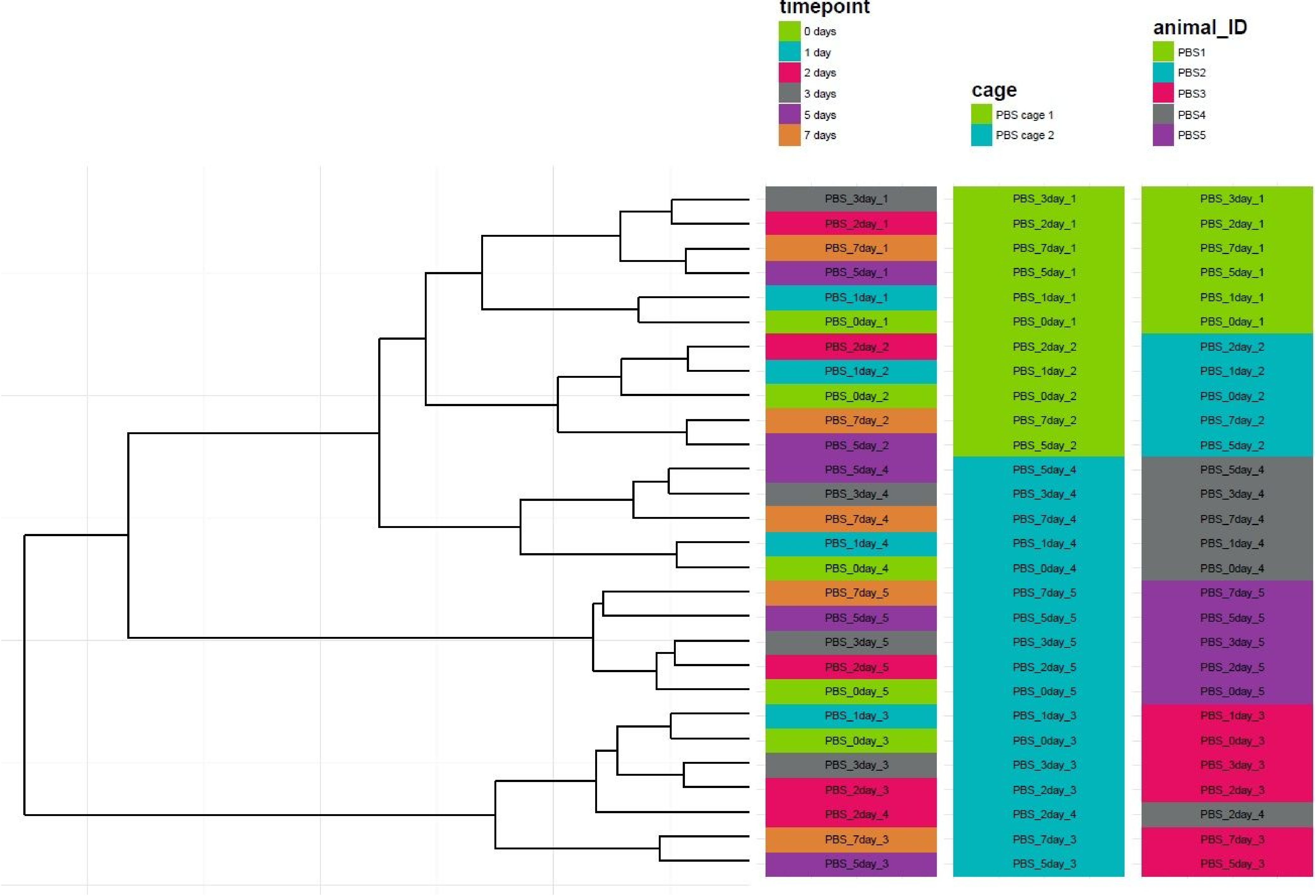

Supplementary Figure S6

Hierarchical clustering of PBS-treated samples over time. Dendogram of all microbiota samples extracted from feces of rats intrarectally inoculated with PBS over seven days. Dendograms were created using the Ward 2 method. Samples clustered by cage number and animal ID. Sample name format is: Treatment_DayFecesCollected_AnimalID.

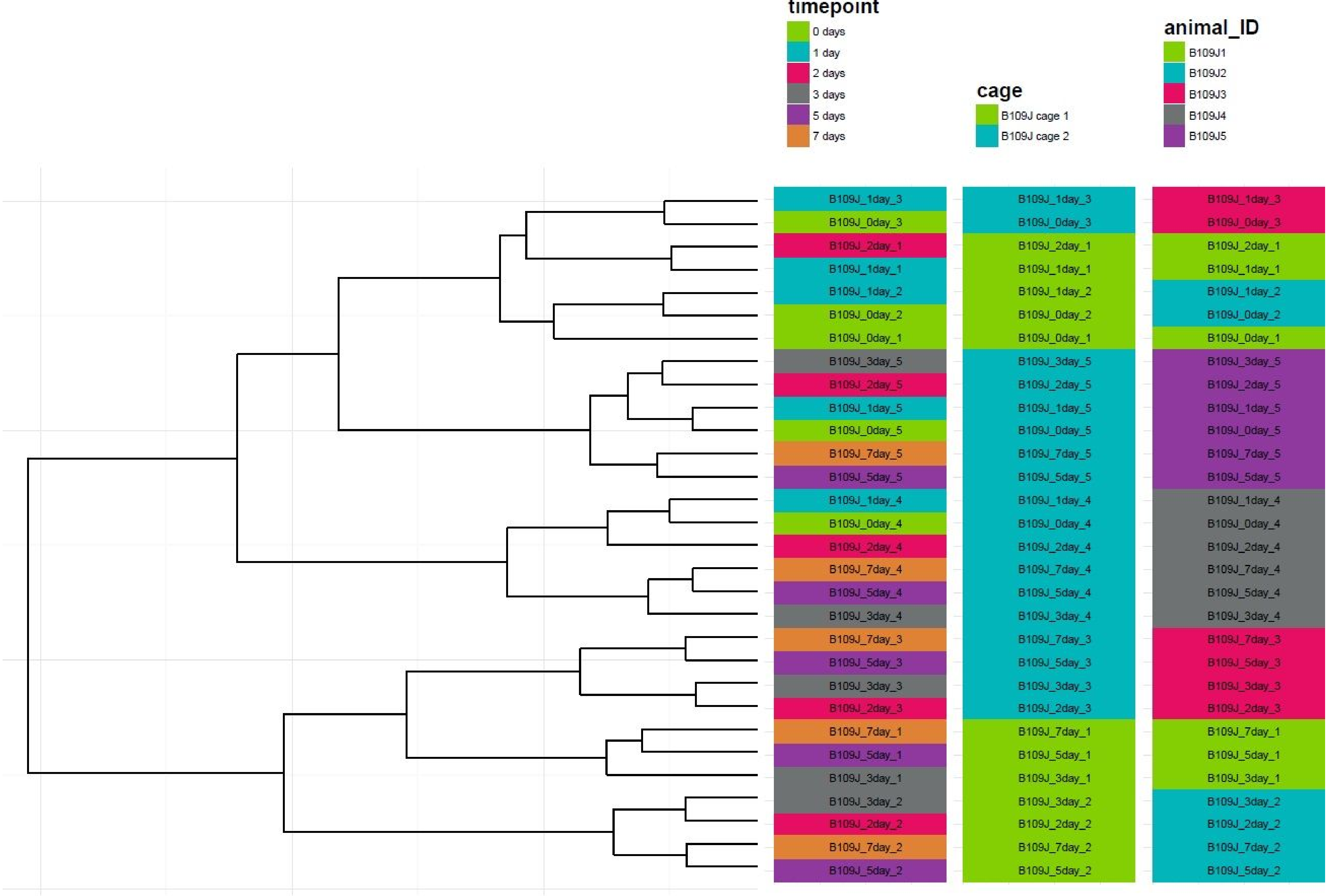

Supplementary Figure S7

Hierarchical clustering of *B. bacteriovorus*-treated samples over time. Dendogram of all microbiota samples extracted from feces of rats intrarectally inoculated with *B. bacteriovorus* 109J (B109J) over seven days. Dendograms were created using the Ward 2 method. Samples formed two main clusters by time point (1st cluster at earlier time points 0-2 days, 2nd cluster at 3-7 days). Samples further sub-clustered according to cage and animal ID. Sample name format is: Treatment_DayFecesCollected_AnimalID.

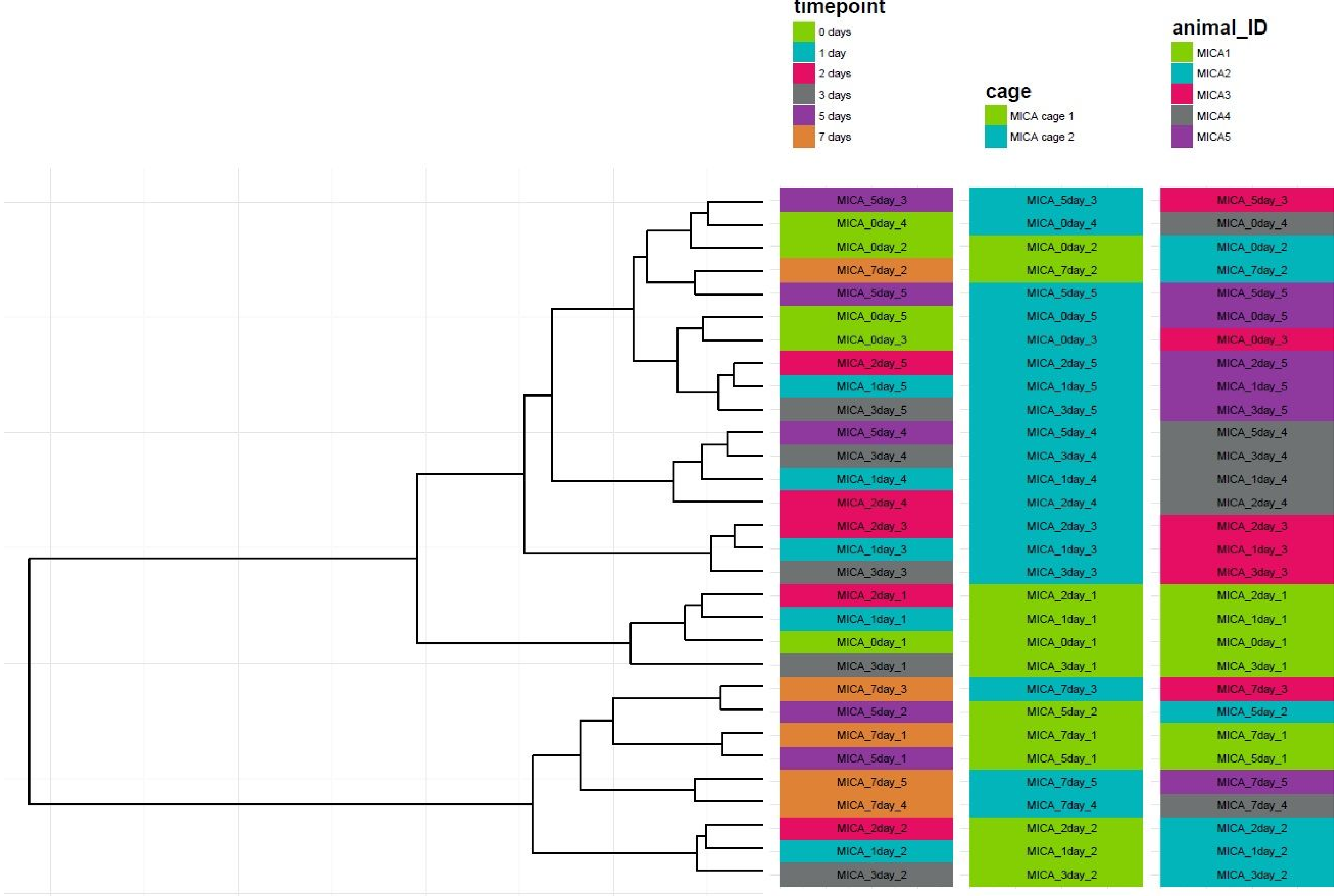

Supplementary Figure S8

Hierarchical clustering of *M. aeruginosavorus*-treated samples over time. Dendogram of all microbiota samples extracted from feces of rats intrarectally inoculated with *M. aeruginosavorus* (MICA) over seven days. Dendograms were created using the Ward 2 method. Clustering patterns appeared to be driven by animal ID. Sample name format is: Treatment_DayFecesCollected_AnimalID.

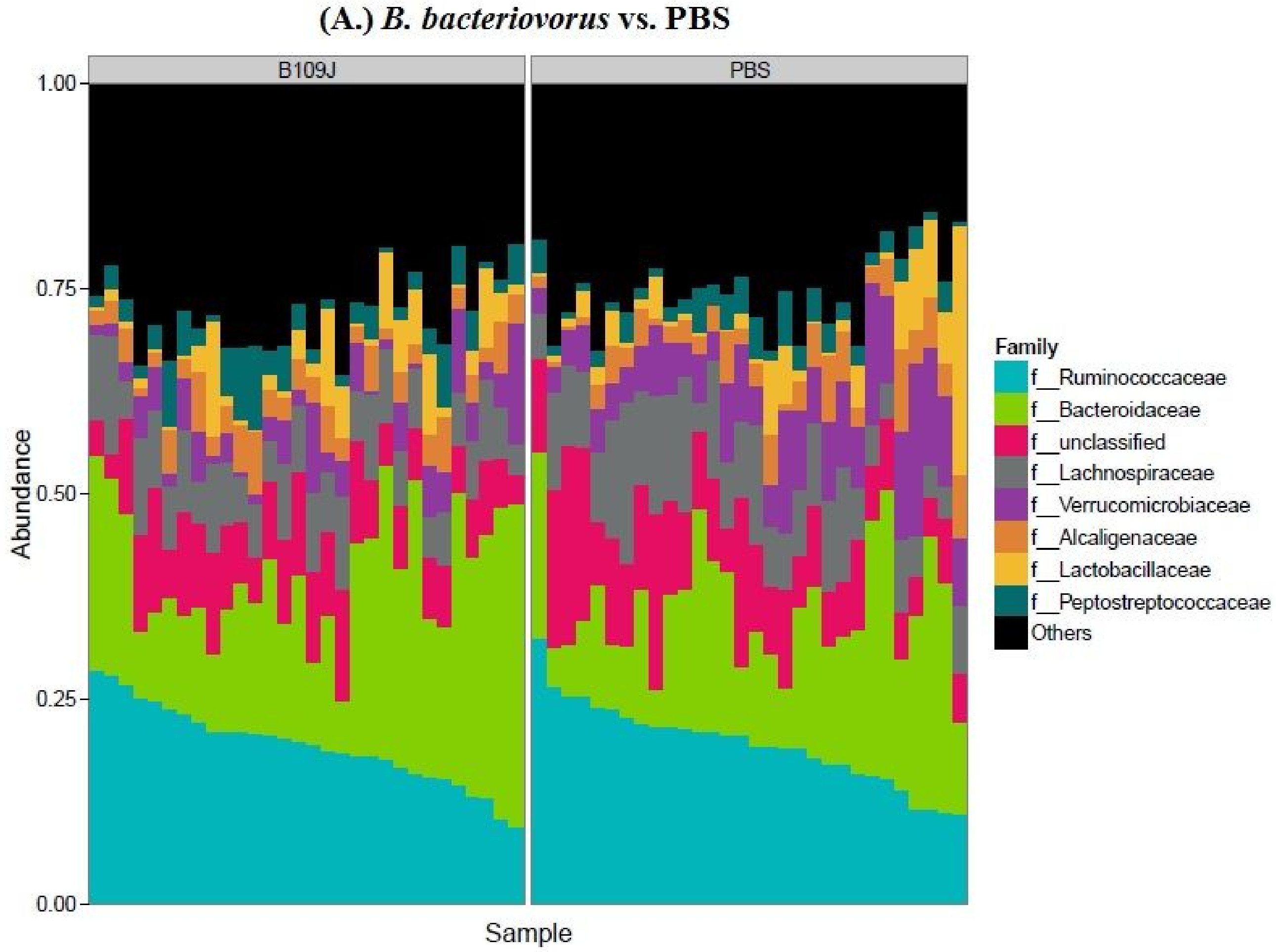

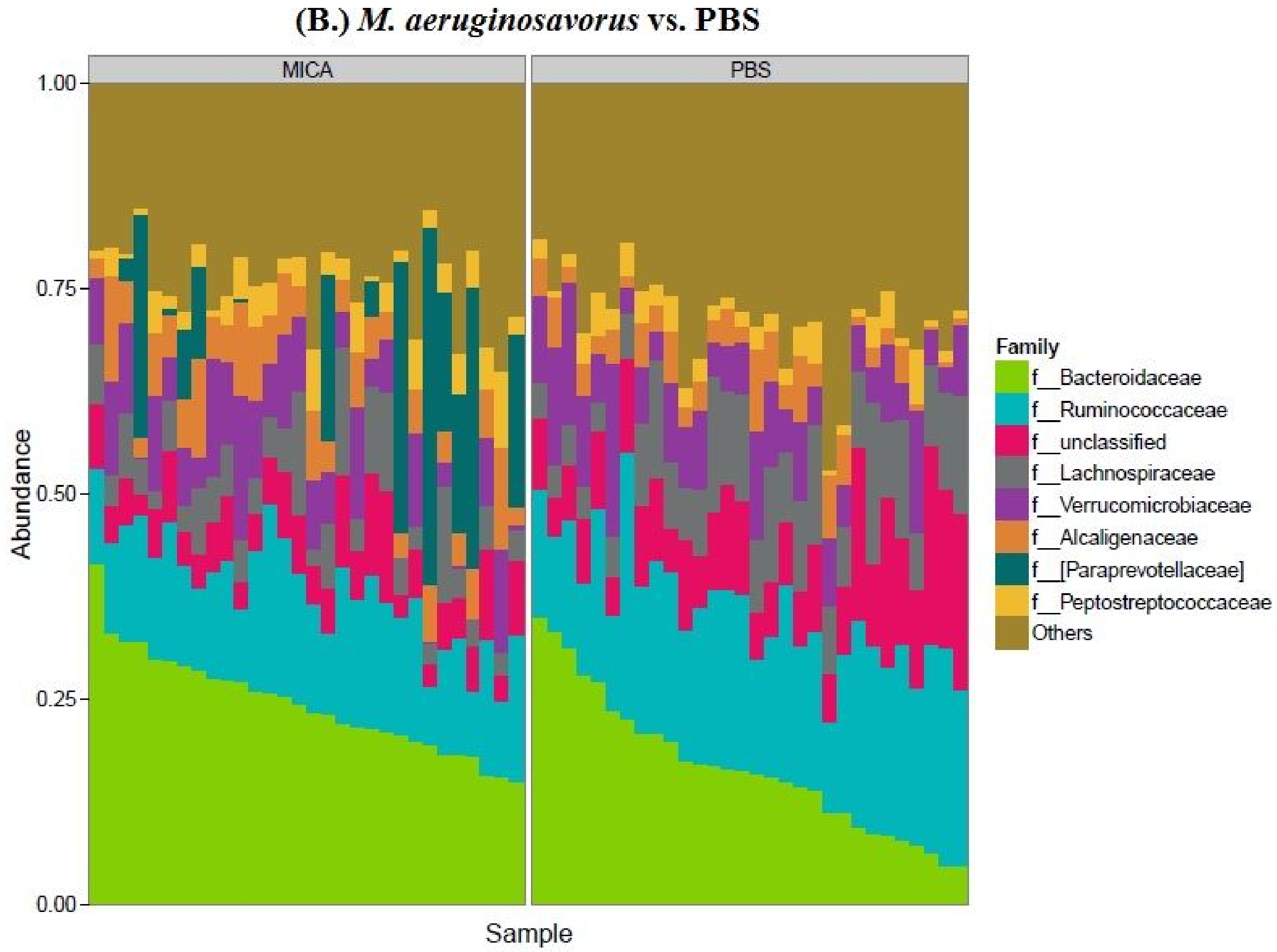

Supplementary Figure S9

Proportional abundance relative to PBS control. Relative abundances of most abundant microbial populations by family in all feces collected from rats intrarectally inoculated with (A) *B. bacteriovorus* (B109J) or (B) *M. aeruginosavorus* (MICA) compared to PBS. Plots show the most abundant taxa at the Family level.

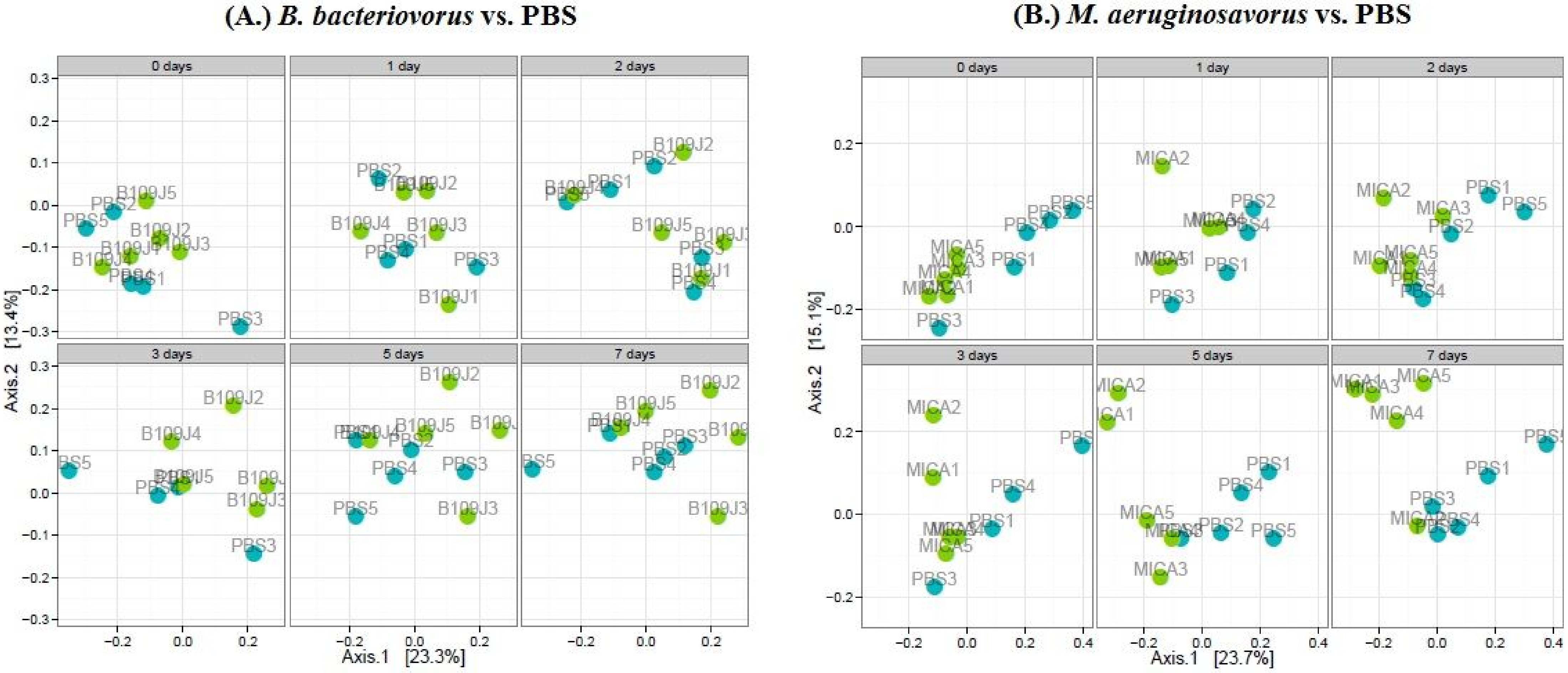

<u>Supplementary Figure S10</u>

Weighted ordination analysis using abundance of predatory bacteria-treated samples vs. PBS control. Dimensional reduction of the Bray-Curtis distance between samples tested from feces collected from rats intrarectally inoculated with (A) *B. bacteriovorus* (B109J) vs. PBS, or (B) *M. aeruginosavorus* (MICA) vs. PBS over seven days, using the principal coordinate analysis (PCoA) ordination method.

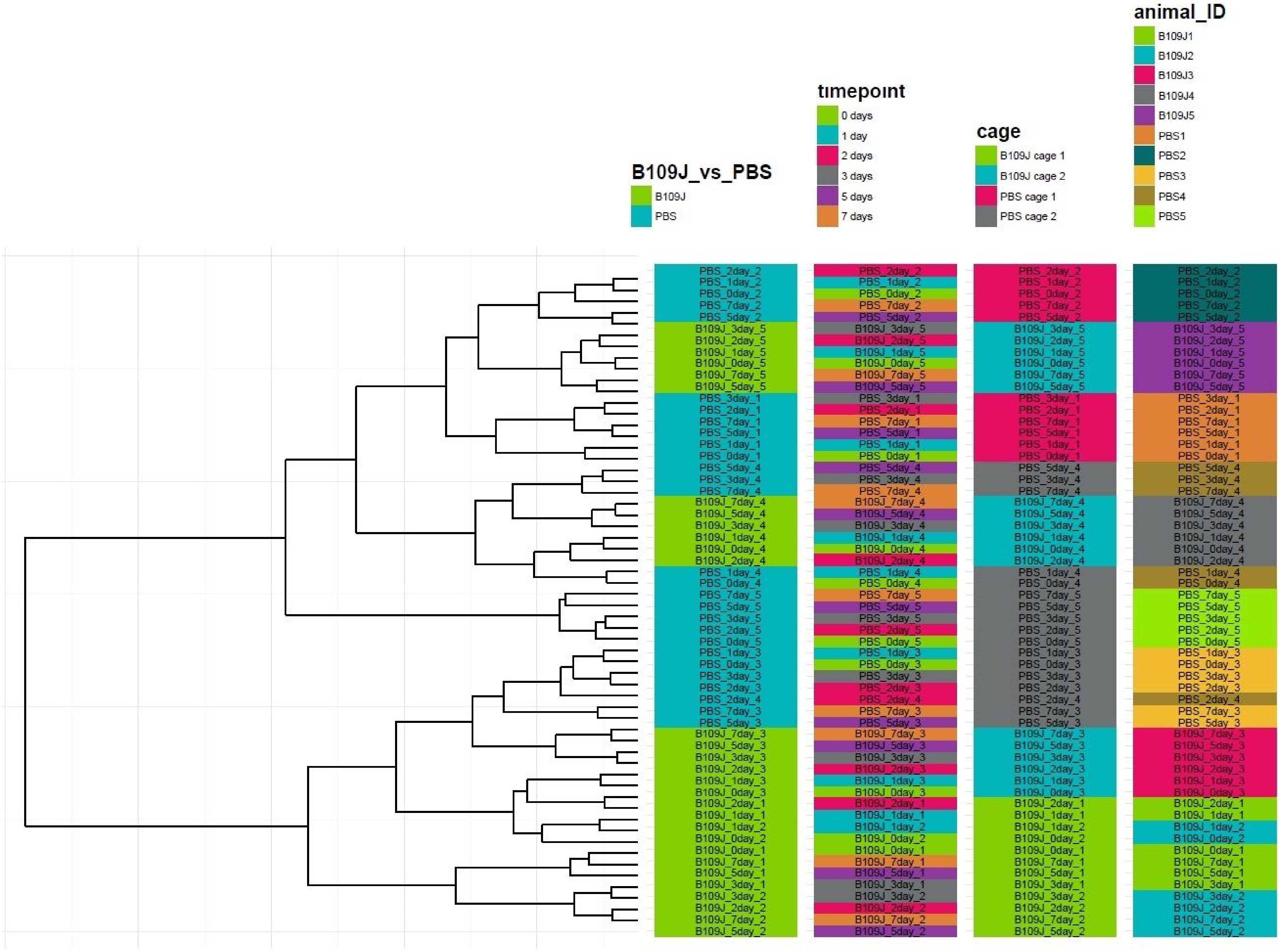

Supplementary Figure S11

Hierarchical clustering of *B. bacteriovorus*-treated samples vs. PBS control. Dendogram of microbiota samples extracted from feces of rats intrarectally inoculated with *B. bacteriovorus* 109J (B109J) vs. PBS over seven days. Dendograms were created using the Ward 2 method. Samples clustered by animal ID, cage number, and treatment group. Sample name format is: Treatment_DayFecesCollected_AnimalID.

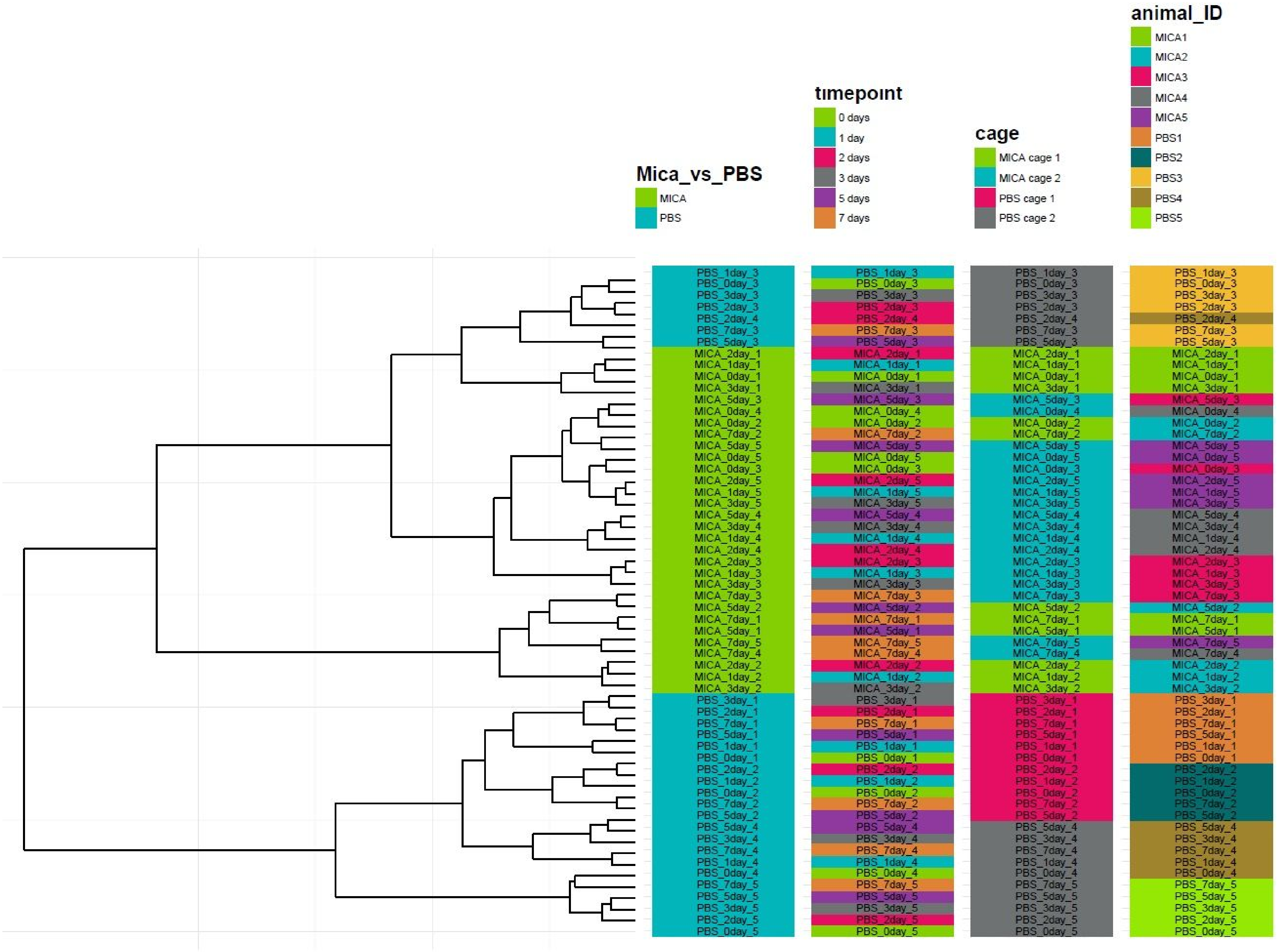

<u>**Supplementary Figure S12**</u>

Hierarchical clustering of *M. aeruginosavorus*-treated samples vs. PBS control. Dendogram of microbiota samples extracted from feces of rats intrarectally inoculated with *M. aeruginosavorus* (MICA) vs. PBS over seven days. Dendograms were created using the Ward 2 method. Samples clustered by treatment group, and further sub-clustered according to cage number and animal ID. Sample name format is: Treatment_DayFecesCollected_AnimalID.
